# Disease Burden of Type 2 Diabetes Among Young Adults in Asia: An Analysis From the Global Burden of Disease Study 2021

**DOI:** 10.1155/jdr/5521613

**Published:** 2025-09-30

**Authors:** Ruoting Wang, Gregory Y. H. Lip, Yingxin Liu, Ningyu Qi, Xuerui Bai, Lehana Thabane, Guowei Li, Harriette G. C. Van Spall

**Affiliations:** ^1^Center for Clinical Epidemiology and Methodology (CCEM), The Affiliated Guangdong Second Provincial General Hospital of Jinan University, Guangzhou, China; ^2^Liverpool Centre for Cardiovascular Science at University of Liverpool, Liverpool John Moores University and Liverpool Heart & Chest Hospital, Liverpool, UK; ^3^Department of Clinical Medicine, Danish Center for Clinical Health Services Research, Aalborg University, Aalborg, Denmark; ^4^Father Sean O'Sullivan Research Centre, St. Joseph's Healthcare Hamilton, Hamilton, Ontario, Canada; ^5^Department of Health Research Methods, Evidence, and Impact (HEI), McMaster University, Hamilton, Ontario, Canada; ^6^Department of Medicine, McMaster University, Hamilton, Ontario, Canada; ^7^Population Health Research Institute, McMaster University, Hamilton, Ontario, Canada

**Keywords:** disability-adjusted life years, global disease burden, incidence, Type 2 diabetes mellitus, young adults

## Abstract

**Background:**

Asia is experiencing the most significant and rapid increase in Type 2 diabetes mellitus (T2DM) globally. Previous studies have indicated a trend toward early onset of T2DM in this region. However, the burden of T2DM among young adults in Asia remains unclear. This study is aimed at exploring the burden of T2DM and its attributable risk factors in Asian adults aged 15–39 years from 1990 to 2021, which could generate insights into our further understanding and thus prevention and management of T2DM in this population.

**Methods:**

Data from Global Burden of Disease 2021 was used to estimate the trends in age-standardized incidence, disability-adjusted life years (DALYs), and mortality of T2DM among young adults in Asia from 1990 to 2021. We analyzed the association between country development level and T2DM burden and investigated the attributable risk factors for T2DM in 2021.

**Results:**

In 2021, total T2DM incidence, prevalence, DALYs, and deaths in Asian young adults were estimated to be 4.55, 59.69, 4.14 million, and 12,769, respectively. There was a consistent annual increase in age-standardized incidence (average annual percentage change [AAPC]: 2.39%) and DALYs (AAPC: 2.08%) and a fluctuating temporal trend in mortality (AAPC: 0.34%) between 1990 and 2021. Incidence and DALYs were higher in males than females in most age groups, although females under 20 years experienced higher DALYs compared to males. Mortality was higher in females before 2007 but lower thereafter. In both males and females, high body mass index was by far the primary attributable risk factor, accounting for 54.56% of T2DM DALYs overall.

**Conclusion:**

The burden of T2DM has increased among young adults in Asia, particularly among females under 20 years. Prevention and treatment of obesity should be prioritized to reduce the burden of T2DM in this population in Asia.

## 1. Introduction

Diabetes mellitus (DM) is a global public health concern. In 2021, over 529 million people were living with DM, and this number is projected to increase to 1.31 billion by 2050 [1]. Type 2 diabetes mellitus (T2DM) accounts for 90%–95% of DM cases and has grown in prevalence. Notably, approximately 60% of people with T2DM are in Asia, which faces the largest and fastest increase in T2DM globally [2–4]. This epidemiological data aligns with Asia's demographic dominance, where its population size of 4.78 billion accounts for 60% of the world's population [5].

Rapid economic growth in Asia has resulted in lifestyle changes including high glycemic index diets, increased smoking and alcohol consumption, and reduced physical activity [6, 7]. These changes have increased the burden of chronic diseases, especially obesity and T2DM. For example, in 2021, China was reported to have the largest number of T2DM cases worldwide, estimated at 141 million; it also had consumption of diets with the highest glycemic index [7]. Young adults are particularly susceptible to modern lifestyles, with an alarming global increase of T2DM cases from 2013 to 2021 [2, 8]. According to the Joint Asia Diabetes Evaluation (JADE) Program, over 18% of the individuals with T2DM were diagnosed below 40 years [9].

The disease burden of T2DM among Asian young adults has not been fully explored, but could generate insights into our further understanding and thus prevention and management of T2DM in this population. Therefore, in this study, we used the Global Burden of Disease (GBD) 2021 data to investigate the burden of T2DM among young adults aged 15–39 years in Asia from 1990 to 2021.

## 2. Methods

### 2.1. Data Source

The GBD study 2021, coordinated by the Institute for Health Metrics and Evaluation (IHME), provides a comprehensive assessment of mortality, incidence, prevalence, years of life lost (YLLs), years lived with disability (YLDs), and disability-adjusted life years (DALYs) attributable to 371 diseases or injuries and 88 risk factors, across 204 countries and territories from 1990 to 2021 [10, 11]. This extensive dataset is derived from a multitude of sources, including population censuses, civil registration and vital statistics, household surveys, disease registries, air pollution monitors, health service usage records, disease notifications, and satellite imaging [11]. Detailed methodologies of the GBD 2021 study have been documented elsewhere [11].

In this study, data on the absolute numbers, non–age-standardized incidence/prevalence, DALYs, and mortality of T2DM among young adults (aged 15–39 years) in Asia, along with their corresponding 95% uncertainty intervals (UIs), from the Global Health Data Exchange website (http://ghdx.healthdata.org/gbd-results-tool) during the period between 1990 and 2021 were used to assess the disease burden of T2DM among Asian young adults.

### 2.2. Measures of T2DM

DM was characterized by a fasting plasma glucose level of ≥ 126 mg/dL (7 mmol/L) or receiving treatment for diabetes as reported by participants [12]. Type 1 diabetes mellitus (T1DM) referred to DM cases that were treated with insulin, identified through hospital records or diabetes registries, or diagnosed by physicians as T1DM [13]. The burden of DM and T1DM was assessed using DisMod-MR 2.1, a Bayesian metaregression tool. The criteria for diagnosing T2DM in the methodological sections were not specific in the GBD study [13]. Therefore, there were no direct model estimates for T2DM, and its burden was estimated indirectly by subtracting the T1DM burden from the overall DM burden [13].

The incidence and prevalence of DM overall and T1DM were estimated using a comprehensive modeling approach that integrated data from systematic reviews, survey data, and longitudinal studies. This approach included the use of DisMod-MR 2.1 to account for severity distributions, disability weights, and adjustments for comorbidities [11, 14]. YLDs were calculated by using diabetic sequelae (neuropathy, diabetic foot, lower limb amputation, and vision loss due to retinopathy) and corresponding disability weights [1]. To estimate YLLs, the number of deaths was multiplied by the remaining life expectancy at the time of death. Subsequently, DALYs were calculated by summing the YLLs and prevalence-based YLDs, thereby capturing both premature mortality and disability from T2DM-specific complications [14]. Deaths due to T2DM were documented according to the 10th revision of the International Classification of Diseases (ICD-10). Afterwards, T2DM mortality was estimated using a Bayesian hierarchical Cause of Death Ensemble model [14]. More details regarding the estimation of incidence, DALYs, and mortality of T2DM could be found elsewhere [14].

### 2.3. Attributable Risk Factors for T2DM

We used the population attributable fraction (PAF) to estimate the attributable burden of T2DM DALYs, where PAF represented the proportion of DALYs that could be prevented if exposure to a risk factor was reduced to the theoretical minimum risk exposure level [14]. There were eight groups of attributable risk factors for T2DM captured in the GBD study, including alcohol consumption, air pollution (including ambient particulate matter pollution and household air pollution from solid fuels), dietary risks (including a diet high in processed meat, a diet high in red meat, a diet high in sugar-sweetened beverages, a diet low in fiber, a diet low in fruits, a diet low in vegetables, and a diet low in whole grains), high body mass index, high fasting plasma glucose, nonoptimal temperature (including high temperature and low temperature), low physical activity, and tobacco (including secondhand smoke and smoking). Given the ongoing debate about the protective effect of moderate alcohol consumption on T2DM, we excluded alcohol consumption from the analysis of attributable risk factors [12]. Furthermore, the high fasting plasma glucose was assumed to have a PAF of 100% for T2DM from the GBD 2021, thereby being excluded from this analysis [10].

### 2.4. Statistical Analyses

Age-standardized incidence, prevalence, DALYs, and mortality with corresponding 95% confidence intervals (CIs) were calculated based on the world standard population [15]. We used the Joinpoint Regression Program (Version 4.9.1.0) to calculate the average annual percentage change (AAPC) with corresponding 95% CIs for age-standardized incidence, DALYs, and mortality of T2DM in Asian young adults aged 15–39 years from 1990 to 2021. The Joinpoint Regression analysis modeled sequential linear segments on a logarithmic scale, with the segments connected at joinpoints where statistically significant changes in the trend over time were observed [16].

We presented results in total and disaggregated by sex, age group (15–19, 20–24, 25–29, 30–34, and 35–39 years), and country/region in Asia. According to the United Nations statistical division, Asia was divided into 48 countries/regions, among which 47 are generally recognized sovereign countries and 1 (the State of Palestine) is a national entity with a special international status (Supporting Information 1: Table S1) [6]. To assess sex differences in age-specific burden of T2DM, we subtracted the age-specific incidence and DALYs of females from males, with a sex difference > 0 indicating higher incidence and DALYs in males [12]. The *Z* test was used to evaluate potential differences in the incidence and DALYs between males and females [17].

We used sociodemographic index (SDI), universal health coverage (UHC), and service capacity/access to analyze the association between country development level and age-standardized incidence (or DALYs). The SDI, ranging from 0 (worst) to 1 (best), was a composite measure of the total fertility rate in women < 25 years, the average education level in individuals aged ≥ 15 years, and the lag-distributed income per capita [18]. UHC denoted that all individuals and communities received the necessary health services without suffering financial hardship, which covered a full range of basic quality health services including health promotion, disease prevention, treatment, rehabilitation, and palliative care [19]. Service capacity/access referred to the ability of a healthcare system to provide necessary health services to the population and how easily individuals could obtain these services [19]. We analyzed the associations between country development level and age-standardized incidence (or DALYs) using an unadjusted linear regression model, where the coefficient with a *p* value < 0.05 indicated a significant relationship [20].

We presented PAF for T2DM in 2021 in descending order and compared the attributable risk factors between males and females. As an exploratory analysis, we calculated the difference in T2DM burden by the age-standardized incidence and DALYs in 2021 minus those in 2019, where a difference > 0 indicated a growing trend from 2019 to 2021. We also explored the association between SDI and the difference in T2DM burden using the unadjusted linear regression model.

Statistical analyses were conducted using R software (Version 4.1.1). A *p* value of less than 0.05 was considered statistically significant.

## 3. Results

### 3.1. T2DM Burden in 2021 and Its Temporal Trend From 1990 to 2021 Among Young Adults in Asia

**Figure 1:**
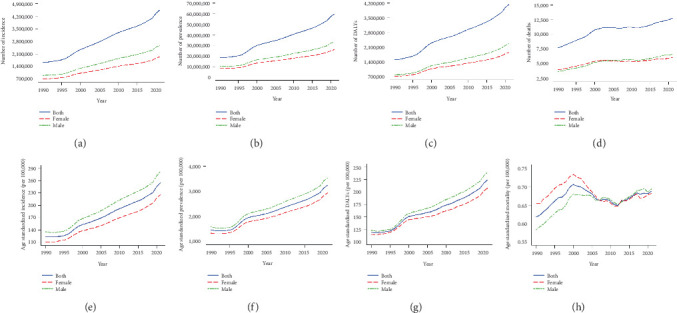
Temporal trends in the burden of T2DM between 1990 and 2021 among young adults in Asia. (a) Number of incidence. (b) Number of prevalence. (c) Number of DALYs. (d) Number of deaths. (e) Age-standardized incidence. (f) Age-standardized prevalence. (g) Age-standardized DALYs. (h) Age-standardized mortality.

In 2021, total T2DM incidence, prevalence, DALYs, and deaths in Asian young adults were estimated to be 4.55 million (95% UI: 3.86, 5.24), 59.69 million (95% UI: 52.31, 68.71), 4.14 million (95% UI: 2.94, 5.71), and 12,769 (95% UI: 11,274, 14,555), respectively (Figures 1a, 1b, 1c, and 1d). The age-standardized incidence, prevalence, DALYs, and mortality per 100,000 population were 254.89 (95% CI: 184.38, 333.26), 798.75 (592.81, 1031.39), 224.10 (95% CI: 157.55, 308.36), and 0.69 (95% CI: 0.60, 0.80), respectively (Figures 1e, 1f, 1g, and 1h and Supporting Information 1: Table S2). These values were consistently higher in males than females in 2021.

**Figure 2:**
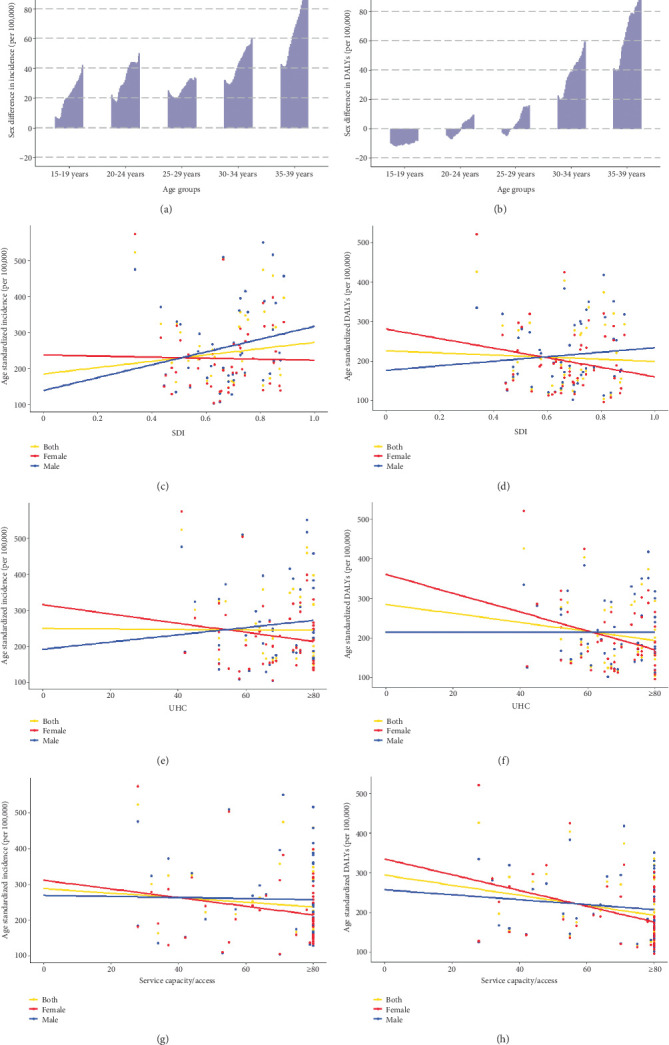
Sex difference in age-specific incidence and DALY rate from 1990 to 2021, and correlation between country development level and age-standardized incidence and DALYs of T2DM in 2021. The sex difference was calculated by the age-specific incidence (or DALYs) in males minus that in females, with a sex difference > 0 indicating a higher incidence (or DALYs) in males. (a) Sex difference in age-specific incidence. (b) Sex difference in age-specific DALYs. (c) Correlation between SDI and age-standardized incidence. (d) Correlation between SDI and age-standardized DALYs. (e) Correlation between UHC and age-standardized incidence. (f) Correlation between UHC and age-standardized DALYs. (g) Correlation between service capacity/access and age-standardized incidence. (h) Correlation between service capacity/access and age-standardized standardized DALYs.

From 1990 to 2021, there was a significant increase in age-standardized incidence (AAPC = 2.39%, 95% CI: 2.25, 2.52) and DALYs (AAPC = 2.08%, 95% CI: 1.95, 2.22) (Supporting Information 1: Table S2). In contrast, a fluctuation was found in age-standardized mortality with an AAPC of 0.34% (95% CI: 0.22, 0.45; Supporting Information 1: Table S2). Young male adults had consistently higher T2DM incidence and DALYs than females over the past three decades across most age groups (Supporting Information 1: Figure S1 and Supporting Information 1: Table S3), and they also faced higher AAPCs in incidence and DALYs than females. The exception to this pattern was among adults aged < 20 years, where females consistently experienced higher DALYs than males over the past three decades (Figure 2). The sex differences in T2DM burden tended to increase over time (Figure 2). However, females had a higher mortality rate than males between 1990 and 2007, but a lower mortality thereafter (Figure 1).

**Figure 3:**
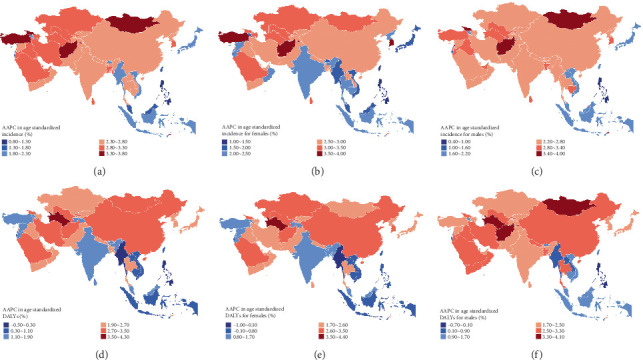
Geographic map of average annual percentage change (AAPC) in age-standardized incidence and DALYs of T2DM from 1990 to 2021 among young adults in Asia. (a) AAPC in incidence for all young adults. (b) AAPC in incidence for females. (c) AAPC in incidence for males. (d) AAPC in DALYs for all young adults. (e) AAPC in DALYs for females. (f) AAPC in DALYs for males.

All 48 countries/regions had an AAPC > 0 in age-standardized T2DM incidence, with the greatest increase found in Brunei Darussalam (Figure 3 and Supporting Information 1: Table S4). Except for Myanmar, the Philippines, and Maldives, all the other countries/regions experienced an increase in age-standardized DALYs from 1990 to 2021. Turkmenistan had the greatest increase in DALYs over time (Figure 3 and Supporting Information 1: Table S4). In 2021, the highest age-standardized incidence and DALYs were observed in Afghanistan (Supporting Information 1: Table S4). Similar trends in T2DM burden were found when data were separated by sex (Figures 3, 3, 3, and 3).

### 3.2. Association Between Country Development Level and T2DM Burden in 2021

Countries/regions with higher SDI experienced higher incidence but lower DALYs when compared to those with lower SDI (Figure 2). UHC and service capacity/access were associated with decreased T2DM burden, yet the relationship was not significant (Figures 2, 2, 2, and 2; *p* values > 0.05). Similar associations were observed in analyses separated by sex and age group (Figures 2, 2, 2, 2, 2, and 2 and Supporting Information 1: Figure S2).

### 3.3. Attributable Risk Factors for DALYs of T2DM in 2021

**Figure 4:**
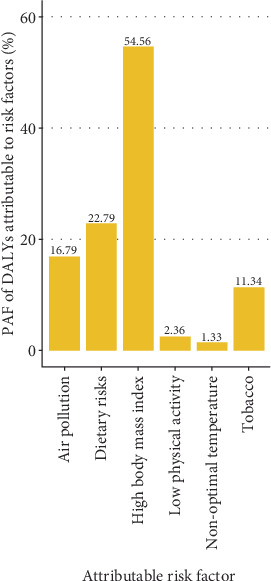
Population attributable fraction (PAF) of T2DM DALYs attributable to risk factors in 2021 among young adults in Asia.

The dominant risk factor for T2DM DALYs was high body mass index in 2021 (PAF = 54.56%), followed by dietary factors (PAF = 22.79%, including diet high in processed meat, diet high in red meat, diet high in sugar-sweetened beverages, diet low in fiber, diet low in fruits, diet low in vegetables, and diet low in whole grains), and air pollution (PAF = 16.79%, including ambient particulate matter pollution and household air pollution from solid fuels) (Figure 4 and Supporting Information 1: Figure S3A). The top five factors in females were high body mass index, ambient particulate matter pollution, secondhand smoke, household air pollution from solid fuels, and diet low in whole grains. The top factors in males included high body mass index, ambient particulate matter pollution, smoking, diet low in whole grains, and diet low in fruits (Supporting Information 1: Figure S3B). Females tended to be more susceptible to secondhand smoke (PAF: 7.14% vs. 4.29%) and household air pollution from solid fuels (6.33% vs. 5.02%) when compared with males (Supporting Information 1: Figure S4). By contrast, males had a substantially higher PAF of smoking (10.38% vs. 0.81%).

### 3.4. Difference in T2DM Burden Between 2019 and 2021

The vast majority of Asian countries/regions experienced an increased T2DM burden from 2019 to 2021 (Supporting Information 1: Figure S5 and Supporting Information 1: Table S5). The Republic of Korea was unique in having a high SDI (0.89) and yet experiencing an increase in T2DM burden (difference in incidence and DALYs: 42.17 and 34.29 per 100,000 population, respectively). The largest increase in incidence during the 3-year period was observed in Indonesia (76.93), while Yemen had the largest increase in DALYs (44.02).

## 4. Discussion

In this study of the GBD 2021 dataset, we demonstrate that Asian young adults experienced the highest incidence and prevalence of T2DM globally in 2019 to 2021 (Supporting Information 1: Figure S6). There were consistent increases in age-standardized incidence and DALYs, with a fluctuating trend in mortality. Overall, males exhibited a higher disease burden than females; however, females aged < 20 years experienced higher DALYs compared with males over the past three decades. Among attributable risk factors for T2DM DALY, a high body mass index was the leading cause in both males and females.

Rapid urbanization in many Asian countries has led to significant lifestyle changes characterized by sedentary behaviors and unhealthy dietary habits, along with heightened environmental pollution [21]. These changes could explain the increasing obesity and T2DM in young adults who are especially susceptible populations to modern lifestyles [22]. In Asia, traditional diets have shifted toward ultraprocessed foods, low fruits and whole grains, high-calorie foods with unhealthy fats, and sugar-sweetened beverages, especially among young adults [23]. Chinese diets had the highest glycemic index worldwide, partly explaining the largest number of T2DM cases in Chinese young adults [7]. Moreover, participants were found to have a higher genetic predisposition to developing T2DM at a younger age in India, which has the largest population in the world [24]. Notably, given the rural–urban disparities in access to healthcare in India, the T2DM burden was probably underestimated in young adults in rural areas [25]. A comprehensive strategy involving food and economic policy changes, public health initiatives, education as a step to improving socioeconomic status, and healthy lifestyles is urgently needed to control and manage the T2DM burden in this population. Environmental pollution, increasingly recognized as a risk factor for cardiometabolic disease, must receive greater priority [26].

Age is recognized as a modifying factor affecting sex differences in T2DM burden among young adults [12]. An overall higher T2DM burden was observed in young males compared to females. Males may be more susceptible to T2DM at a lower BMI due to a combination of biological and lifestyle patterns [27]. Females have a higher percentage of body fat and tend to store subcutaneous fat, which is less metabolically harmful compared to the visceral fat more commonly seen in males [27]. Although males are more likely to develop T2DM, females with T2DM tend to experience more severe complications [28]. Prior to the early 21st century, cultural and societal norms often placed greater emphasis on addressing males' health needs, leaving females more susceptible to underdiagnosis and harmful effects of chronic diseases such as T2DM [29]. It is also possible that adverse effects from dysglycemia accrue at a lower threshold in females than males; the threshold for diagnosis of diabetes in males versus females has been called into question [30]. We found that females aged under 20 years consistently had higher DALYs from 1990 to 2021. One possible reason may be the hormonal changes and cessation of physical activity or sports during female puberty, which was reported to associate with overweight and thus insulin resistance [31]. The higher T2DM burden in girls aged < 20 years may be also due to social determinants such as access to and engagement in education, sports, and career-related activities rather than household labor activities [32]. Unlike previous studies providing data on the T2DM burden globally [33] or on adult populations [34], our study highlighted the sex-specific intervention measures or public health strategies in Asia among young adults, particularly for females under 20 years of age. The World Health Organization (WHO) and national societies have begun to focus on these sex disparities, generating guidelines and policies to address them [35]. During this same period, changes in lifestyle had a disproportionate impact on males, contributing to higher rates of T2DM complications and mortality [36]. These trends may help explain why the mortality rate from T2DM was initially higher in females but later became lower than in males.

As country SDI increased, the age-standardized T2DM incidence increased, but DALYs decreased. A higher SDI often correlates with better access to healthcare, leading to increased diagnosis rates of T2DM [37]. Additionally, in high SDI countries, there is typically a shift from traditional to more urban lifestyles, with reduced physical activity and increased consumption of Western diets characterized by calorie-dense, processed foods, which can contribute to a higher incidence of T2DM [36]. This improved detection, coupled with more effective management of the disease, contributes to the reduction in DALYs or years of health lost; complications and mortality rates decrease [37]. Conversely, in low SDI countries or regions, young adults are more likely to remain undiagnosed due to limited access to healthcare, resulting in a seemingly lower T2DM incidence [38]. However, the lack of timely diagnosis and treatment contributes to a higher burden of disease, reflected in increased DALYs and premature death [38]. Therefore, promoting economic development and optimizing healthcare resource allocation in countries with lower SDI are essential strategies to address these disparities.

Obesity was reported as the primary cause of T2DM in adolescents and young adults [39], in line with our results. This underscores the fundamental importance of preventing overweight/obesity through comprehensive public health strategies. Priority should be given to promoting lifestyle modifications, including healthy dietary patterns, increased physical activity, and education, particularly targeting young populations. Early intervention, engaging parents, schools, and communities to achieve widespread “buy-in,” is critical for sustainable long-term prevention and amelioration of the T2DM burden in Asia [40]. While intensive lifestyle interventions focused on weight reduction remain the cornerstone for mitigating and even reversing T2DM [40], the pharmacological landscape is evolving. The uptake of glucagon-like peptide-1 receptor agonists (GLP-1 RAs) for obesity management is growing in Asia, though significant challenges related to availability, cultural perceptions, and especially cost persist [41]. The identification of high BMI as the primary attributable risk factor reinforces the critical role of obesity prevention and management in reducing the T2DM burden. Previous research found that secondhand smoke exposure in public areas became more prevalent among adolescents aged 12–16, especially in girls [42, 43]. We also observed that females were more susceptible to secondhand smoke compared to males, underscoring the need for smoking cessation in males. Notably, the household air pollution from solid fuels was a risk factor for T2DM in Asian young females, which may link to their prolonged exposure to poor cooking conditions with fossil fuels and inadequate ventilation in Asia [22, 44].

Higher SDI countries/regions experienced a relatively small increase in burden largely due to their healthcare resources, public health measures, and access to health information and promotion [45]. Despite its high SDI, the Republic of Korea had a significant strain on its healthcare system during the early outbreak, which resulted in delays and disruptions in routine medical care and adequate prevention and management of T2DM [46]. Nevertheless, results from this ecological and exploratory analysis were hypothesis-generating and could not explain the causal link between COVID-19 and change in T2DM burden in individual country/region.

### 4.1. Strengths and Limitations

Our study evaluated the disease burden of T2DM among young adults in Asia, which could guide evidence-based policy-making for T2DM prevention and control. Several limitations need to be acknowledged. The diagnosis of T1DM relies on insulin treatment, yet studies have shown that some young adults misdiagnosed with T1DM may actually have T2DM [47, 48]. This misclassification could affect data interpretation, as insulin-treated patients might include those who are misdiagnosed, those experiencing rapid vascular complications, or those struggling with glycemic control. However, since relatively few patients were on insulin, we consider that the impact of misdiagnosis on our results is limited. Because the estimates of overall DM were not from multiple fasting blood glucose measurements or oral glucose tolerance tests, the T2DM estimates may be suboptimal. In this study, we emphasized the incidence of T2DM (rather than prevalence) because data on the incidence could specifically provide a measure of new cases within a period to understand the disease patterns, emerging trends, and risk factors and thus to guide the contemporary evidence-based policy-making. Of note, the exact onset of T2DM may be unclear given the T2DM as a chronic condition. However, we observed the consistent temporal trends and disease patterns between incidence and prevalence-based DALYs, partially supporting our findings based on T2DM incidence. In addition, the burden of T2DM in countries/regions with a low SDI may be underestimated due to data and diagnostic gaps. We did not estimate T2DM burden in participants aged < 15 years because data on this group were unavailable in GBD 2021 [49]. Furthermore, this analysis relied on estimated summary data from the GBD, and individual participant data were unavailable; thus, other comorbidities could not be adjusted for in the analyses.

## 5. Conclusion

The elevated burden of T2DM among young adults in Asia increasingly becomes a significant public health concern, and females aged < 20 years require specific attention for prevention and intervention measures. Effective weight control and management are ultimately important to reduce the burden of T2DM in this population in Asia.

## Data Availability

All data based on GBD 2021 in this study are publicly available from the Global Health Data Exchange website (http://ghdx.healthdata.org/gbd-results-tool).

## Supporting Information

Supporting Information 1 Table S1: List of countries or regions in Asia as per the United Nations Statistical Division. Table S2: Age-standardized incidence, DALYs, and mortality of T2DM and AAPC among young adults in Asia∗. Table S3: Age-standardized incidence and DALYs of T2DM and AAPC among young adults by country/region in Asia∗. Table S4: AAPC for age-standardized incidence and DALYs from 1990 to 2021 among Asian young adults separated by sex and age group. Table S5: Age-standardized incidence and DALYs of T2DM and AAPC among young adults by country/region in Asia from 2019 to 2021. Figure S1: Age-specific incidence and DALYs in young adult females and males from 1990 to 2021. (A) Age-specific incidence and (B) age-specific DALYs. Figure S2: Correlation between the sociodemographic index (SDI) (universal health coverage [UHC] or service capacity/access) and age-standardized incidence (or DALYs) of T2DM among young adults in Asia in 2021 across five age groups. (A) Correlation between SDI and age-standardized incidence, (B) correlation between SDI and age-standardized DALYs, (C) correlation between UHC and age-standardized incidence, (D) correlation between UHC and age-standardized DALYs, (E) correlation between service capacity/access and age-standardized incidence, and (F) correlation between service capacity/access and age-standardized DALYs. Figure S3: Population attributable fraction (PAF) of T2DM DALYs attributable to 15 risk factors among young adults in Asia and separated by sex in 2021. (A) For both sexes and (B) separated by sex. Figure S4: Population attributable fraction (PAF) of T2DM DALYs attributable to 15 risk factors in 2021 among young adults in Asia separated by sex. Figure S5: Correlation between the sociodemographic index (SDI) and the difference in T2DM burden between 2021 and 2019. The difference was calculated by the age-standardized incidence (or DALYs) in 2021 minus those in 2019. (A) Difference in age-standardized incidence and (B) difference in age-standardized DALYs. Figure S6: Age-standardized incidence and prevalence among young adults globally from 1990 to 2021 separated by different continents. (A) Incidence and (B) prevalence.

Supporting Information 2 GATHER_checklist. Guidelines for Accurate and Transparent Health Estimates Reporting (GATHER).
