# Supplementary material for: Disease Burden of Type 2 Diabetes Among Young Adults in Asia: An Analysis From the Global Burden of Disease Study 2021
Source: J Diabetes Res. 2025 Sep 30;2025:5521613. doi: 10.1155/jdr/5521613 (PMC12503994; doi:10.1155/jdr/5521613)

Supplemental Contents

STable 1. List of Countries or regions in Asia as per United Nations Statistical Division. 2
STable 2. Age standardized incidence, DALYs and mortality of T2DM and AAPC among young adults in Asia* 4
STable 3. AAPC for age standardized incidence and DALYs from 1990 to 2021 among Asian young adults separated by sex and age group. 5
STable 4. Age standardized incidence and DALYs of T2DM and AAPC among young adults by country/region in Asia* 6
STable 5. Age standardized incidence and DALYs of T2DM and AAPC among young adults by country/region in Asia from 2019 to 2021. 8
SFigure 1. Age specific incidence and DALYs in young adult females and males from 1990 to 2021. (A) age specific incidence; (B) age specific DALYs 11
SFigure 2. Correlation between socio-demographic index (SDI) (universal health coverage [UHC], or service capacity/access) and age standardized incidence (or DALYs) of T2DM among young adults in Asia in 2021 across five age groups. (A) correlation between SDI and age standardized incidence; (B) correlation between SDI and age standardized DALYs; (C) correlation between UHC and age standardized incidence; (D) correlation between UHC and age standardized DALYs; (E) correlation between service capacity/access and age standardized incidence; (F) correlation between service capacity/access and age standardized DALYs 12
SFigure 3. Population attributable fraction (PAF) of T2DM DALYs attributable to 15 risk factors among young adults in Asia and separated by sex in 2021. (A) for both sex; (B) separated by sex 13
SFigure 4. Population attributable fraction (PAF) of T2DM DALYs attributable to 15 risk factors in 2021 among young adults in Asia separated by sex. 14
SFigure 5. Correlation between the socio-demographic index (SDI) and difference in T2DM burden between 2021 and 2019. The difference was calculated by the age standardized incidence (or DALYs) in 2021 minus those in 2019. (A) difference in age standardized incidence; (B) difference in age standardized DALYs 15
SFigure 6. Age standardized incidence and prevalence among young adults globally from 1990 to 2021 separated by different continents. (A) incidence; (B) prevalence .16

STable 1. List of Countries or regions in Asia as per United Nations Statistical Division.

Sub-region of Asia	Country or region
Central Asia	Kazakhstan
	Kyrgyzstan
	Tajikistan
	Turkmenistan
	Uzbekistan
Eastern Asia	China
	Democratic People's Republic of Korea
	Japan
	Mongolia
	Republic of Korea
	Taiwan (Province of China)
South-eastern Asia	Brunei Darussalam
	Cambodia
	Indonesia
	Lao People's Democratic Republic
	Malaysia
	Myanmar
	Philippines
	Singapore
	Thailand
	Timor-Leste
	Viet Nam
Southern Asia	Afghanistan
	Bangladesh
	Bhutan
	India
	Iran (Islamic Republic of)
	Maldives
	Nepal
	Pakistan
	Sri Lanka
Western Asia	Armenia
	Azerbaijan
	Bahrain
	Cyprus
	Georgia
	Iraq
	Israel

	Jordan
	Kuwait
	Lebanon
	Oman
	Qatar
	Saudi Arabia
	State of Palestine
	Syrian Arab Republic
	Türkiye
	United Arab Emirates
	Yemen

STable 2. Age standardized incidence, DALYs and mortality of T2DM and AAPC among young adults in Asia*

	Age standardized measures in 1990 (per 100 000)			Age standardized measures in 2019 (per 100 000)			Age standardized measures in 2021 (per 100 000)			AAPC (%)		
	**Incidence**	**DALYs**	**Mortality**	**Incidence**	**DALYs**	**Mortality**	**Incidence**	**DALYs**	**Mortality**	**Incidence**	**DALYs**	**Mortality**
**Both**	123.75 (84.02, 171.51)	119.01 (88.06, 158.57)	0.62 (0.53, 0.70)	231.73 (167.70, 303.94)	207.43 (146.71, 284.82)	0.68 (0.60, 0.79)	254.89 (184.38, 333.26)	224.10 (157.55, 308.36)	0.69 (0.60, 0.80)	2.39 (2.25, 2.52)	2.08 (1.95, 2.22)	0.34 (0.22, 0.45)
**Females**	110.85 (75.01, 154.59)	114.79 (86.18, 152.34)	0.65 (0.53, 0.78)	205.75 (147.78, 271.78)	192.47 (137.46, 262.89)	0.67 (0.57, 0.79)	226.10 (163.01, 297.67)	207.86 (147.59, 283.92)	0.68 (0.57, 0.80)	2.36 (2.21, 2.50)	1.97 (1.86, 2.08)	0.15 (0.05, 0.24)
**Males**	136.04 (92.45, 188.36)	122.97 (88.75, 165.60)	0.58 (0.49, 0.69)	256.33 (186.36, 334.80)	221.72 (155.65, 306.10)	0.69 (0.58, 0.84)	282.03 (204.96, 367.35)	239.52 (166.88, 331.77)	0.69 (0.58, 0.84)	2.41 (2.27, 2.55)	2.19 (2.06, 2.32)	0.54 (0.39, 0.69)

*Data shown as point estimates (95% confidence intervals)

DALYs: disability-adjusted life years; AAPC: average annual percentage change

STable 3. AAPC for age standardized incidence and DALYs from 1990 to 2021 among Asian young adults separated by sex and age group.

	**AAPC for age standardized incidence**			**AAPC for age standardized DALYs**		
	**Both**	**Female**	**Male**	**Both**	**Female**	**Male**
**15-19 years**	2.83 (2.53, 3.13)	2.59 (2.28, 2.89)	3.00 (2.69, 3.31)	1.70 (1.51, 1.89)	1.48 (1.25, 1.70)	1.94 (1.77, 2.11)
**20-24 years**	2.72 (2.48, 2.95)	2.72 (2.50, 2.95)	2.73 (2.48, 2.98)	2.21 (2.00, 2.43)	1.95 (1.61, 2.29)	2.43 (2.22, 2.65)
**25-29 years**	2.27 (2.15, 2.38)	2.41 (2.27, 2.56)	2.17 (2.06, 2.28)	2.29 (2.14, 2.45)	2.11 (1.95, 2.26)	2.41 (2.18, 2.65)
**30-34 years**	2.09 (2.04, 2.14)	2.11 (2.06, 2.15)	2.10 (2.06, 2.14)	2.11 (2.00, 2.22)	2.02 (1.90, 2.14)	2.20 (2.08, 2.32)
**35-39 years**	2.11 (2.06, 2.16)	1.99 (1.90, 2.08)	2.20 (2.15, 2.25)	2.01 (1.89, 2.13)	1.94 (1.81, 2.06)	2.05 (1.91, 2.18)

*Data shown as point estimates (95% confidence intervals)

DALYs: disability-adjusted life years; AAPC: average annual percentage change

STable 4. Age standardized incidence and DALYs of T2DM and AAPC among young adults by country/region in Asia*

Location	Age standardized incidence in 2021 (per 100 000)	AAPC for age standardized incidence from 1990 to 2021	Age standardized DALYs in 2021 (per 100 000)	AAPC for age standardized DALYs from 1990 to 2021
Central Asia				
Kazakhstan	247.70 (182.11, 324.63)	2.90 (2.86, 2.94)	178.45 (118.93, 253.02)	2.67 (2.52, 2.81)
Kyrgyzstan	166.69 (118.13, 218.75)	2.75 (2.68, 2.83)	117.32 (79.37, 164.29)	2.22 (2.12, 2.32)
Tajikistan	150.97 (108.37, 199.49)	2.58 (2.50, 2.66)	128.71 (89.64, 174.23)	1.67 (1.41, 1.92)
Turkmenistan	165.44 (119.33, 215.89)	3.27 (3.23, 3.31)	181.59 (137.80, 237.81)	4.02 (3.56, 4.48)
Uzbekistan	199.10 (143.86, 262.02)	3.24 (3.12, 3.35)	165.15 (120.29, 221.19)	2.84 (2.55, 3.13)
Eastern Asia				
China	316.49 (227.21, 418.12)	2.68 (2.36, 3.01)	266.26 (170.28, 385.67)	2.75 (2.37, 3.13)
Democratic People's Republic of Korea	226.06 (163.87, 297.00)	2.44 (2.42, 2.45)	208.54 (139.48, 292.91)	2.01 (1.98, 2.04)
Japan	200.05 (138.17, 274.31)	2.09 (1.93, 2.25)	146.88 (94.96, 214.64)	2.09 (1.92, 2.26)
Mongolia	179.82 (130.33, 235.20)	3.40 (3.35, 3.45)	138.35 (93.40, 196.70)	3.13 (2.89, 3.37)
Republic of Korea	397.91 (289.47, 514.53)	3.30 (3.20, 3.40)	293.55 (193.71, 421.09)	2.54 (2.27, 2.81)
Taiwan (Province of China)	182.01 (133.51, 236.40)	1.74 (1.65, 1.82)	155.48 (106.42, 212.65)	0.94 (0.71, 1.16)
South-eastern Asia				
Brunei Darussalam	475.04 (331.41, 645.55)	3.79 (3.74, 3.85)	373.66 (265.29, 507.24)	1.60 (1.32, 1.88)
Cambodia	129.92 (92.66, 171.04)	2.63 (2.55, 2.71)	155.77 (113.20, 209.74)	0.94 (0.82, 1.07)
Indonesia	137.78 (95.52, 184.12)	1.91 (-0.08, 3.95)	141.68 (108.50, 179.92)	0.88 (0.45, 1.32)
Lao People's Democratic Republic	162.55 (116.44, 213.01)	2.34 (2.31, 2.36)	197.36 (145.05, 263.43)	0.62 (0.56, 0.67)
Malaysia	185.80 (128.94, 252.28)	1.76 (1.49, 2.04)	175.07 (127.16, 236.3)	1.20 (1.00, 1.40)
Myanmar	221.85 (162.33, 290.83)	2.17 (2.11, 2.23)	296.97 (226.06, 391.24)	-0.13 (-0.22, -0.04)
Philippines	109.23 (75.73, 148.27)	0.89 (0.71, 1.07)	190.73 (157.54, 232.05)	-0.12 (-0.40, 0.16)
Singapore	315.27 (223.75, 423.15)	1.68 (1.62, 1.74)	221.35 (142.95, 327.42)	1.91 (1.82, 1.99)
Thailand	148.99 (105.25, 197.87)	2.36 (2.26, 2.46)	200.47 (151.91, 257.71)	2.07 (1.61, 2.53)
Timor-Leste	151.54 (108.08, 200.92)	3.58 (3.55, 3.61)	144.67 (103.43, 194.54)	2.03 (1.75, 2.30)
Viet Nam	104.13 (75.43, 136.19)	1.83 (1.73, 1.94)	121.79 (89.15, 164.68)	0.78 (0.71, 0.84)
Southern Asia				
Afghanistan	524.56 (370.57, 693.62)	3.66 (3.60, 3.71)	425.85 (291.38, 590.75)	3.38 (3.31, 3.45)
Bangladesh	325.86 (236.58, 422.60)	3.01 (2.83, 3.18)	278.25 (190.78, 392.60)	2.26 (2.07, 2.46)
Bhutan	216.72 (156.82, 285.64)	2.12 (2.02, 2.23)	176.31 (120.36, 248.12)	1.45 (1.38, 1.52)
India	263.43 (186.09, 350.83)	2.38 (2.35, 2.41)	205.47 (143.14, 286.02)	1.76 (1.61, 1.92)
Iran (Islamic Republic of)	186.55 (125.72, 258.71)	2.83 (2.67, 2.99)	142.57 (99.49, 197.54)	2.72 (2.45, 2.98)
Maldives	132.78 (93.09, 181.06)	1.90 (1.83, 1.96)	127.41 (89.44, 174.32)	-0.24 (-0.48, 0.00)
Nepal	324.89 (233.49, 428.80)	2.60 (2.43, 2.77)	289.46 (198.20, 403.24)	2.30 (2.22, 2.37)

**Pakistan**	300.82 (212.44, 402.55)	2.54 (2.43, 2.64)	283.20 (203.15, 375.75)	2.40 (2.34, 2.45)
**Sri Lanka**	270.48 (187.22, 359.45)	3.05 (3.01, 3.10)	277.91 (202.32, 377.58)	1.88 (1.34, 2.42)
Western Asia				
**Armenia**	161.21 (111.13, 219.79)	1.96 (1.91, 2.02)	119.36 (83.55, 166.25)	1.31 (0.90, 1.73)
**Azerbaijan**	165.56 (116.79, 219.40)	2.82 (2.79, 2.85)	124.90 (85.64, 174.03)	2.33 (2.03, 2.63)
**Bahrain**	336.28 (230.76, 461.98)	2.99 (2.88, 3.09)	335.96 (247.34, 447.36)	2.47 (2.01, 2.93)
**Cyprus**	173.05 (115.06, 239.07)	2.30 (2.25, 2.34)	114.51 (73.21, 168.20)	1.34 (1.13, 1.56)
**Georgia**	220.88 (156.61, 296.19)	3.40 (3.23, 3.57)	155.50 (106.27, 219.51)	2.93 (2.60, 3.27)
**Iraq**	507.49 (350.04, 691.96)	2.84 (2.72, 2.96)	403.20 (274.22, 560.73)	2.23 (2.11, 2.35)
**Israel**	154.29 (103.00, 213.40)	1.70 (1.65, 1.75)	104.97 (70.54, 149.83)	0.56 (0.42, 0.70)
**Jordan**	357.97 (246.91, 484.95)	2.82 (2.66, 2.98)	270.39 (187.26, 367.84)	1.75 (1.52, 1.97)
**Kuwait**	459.29 (320.35, 618.01)	3.01 (2.90, 3.12)	320.11 (207.36, 456.53)	2.76 (2.18, 3.33)
**Lebanon**	349.04 (241.83, 471.33)	3.09 (3.01, 3.17)	283.31 (188.15, 400.17)	2.24 (2.17, 2.30)
**Oman**	229.15 (156.27, 318.06)	2.50 (2.26, 2.74)	182.12 (127.07, 250.69)	2.58 (2.42, 2.75)
**Qatar**	312.34 (211.35, 431.12)	2.97 (2.91, 3.02)	255.36 (175.57, 349.70)	2.21 (1.83, 2.59)
**Saudi Arabia**	359.02 (250.33, 486.40)	2.84 (2.78, 2.91)	291.81 (202.82, 404.75)	3.00 (2.85, 3.15)
**State of Palestine**	232.16 (161.93, 311.13)	2.68 (2.63, 2.74)	202.51 (146.24, 269.01)	1.65 (1.46, 1.84)
**Syrian Arab Republic**	252.28 (177.08, 337.88)	2.37 (2.34, 2.41)	194.17 (133.46, 273.51)	1.59 (1.49, 1.70)
**Türkiye**	189.06 (136.75, 246.90)	3.49 (3.39, 3.59)	150.65 (103.03, 209.02)	1.47 (1.03, 1.92)
**United Arab Emirates**	220.47 (151.27, 300.89)	2.83 (2.78, 2.87)	159.74 (109.66, 221.45)	2.24 (2.11, 2.37)
**Yemen**	247.70 (182.11, 324.63)	2.90 (2.86, 2.94)	178.45 (118.93, 253.02)	2.67 (2.52, 2.81)

*Data shown as point estimates (95% confidence intervals)

DALYs: disability-adjusted life years; AAPC: average annual percentage change

STable 5. Age standardized incidence and DALYs of T2DM and AAPC among young adults by country/region in Asia from 2019 to 2021.

Location	Age standardized incidence in 2021 (per 100 000)	Age standardized incidence in 2019 (per 100 000)	AAPC for age standardized incidence from 2019 to 2021	Age standardized DALYs in 2021 (per 100 000)	Age standardized DALYs in 2019 (per 100 000)	AAPC for age standardized DALYs from 2019 to 2021
Central Asia						
Kazakhstan	247.70 (182.11, 324.63)	242.14 (173.28, 319.71)	1.14 (-0.38, 2.69)	178.45 (118.93, 253.02)	175.82 (116.28, 251.74)	0.75 (-6.46, 8.52)
Kyrgyzstan	166.69 (118.13, 218.75)	160.73 (115.99, 213.95)	1.84 (-3.49, 7.45)	117.32 (79.37, 164.29)	113.62 (77.45, 160.21)	1.62 (-3.33, 6.83)
Tajikistan	150.97 (108.37, 199.49)	146.32 (103.71, 196.16)	1.58 (0.51, 2.66)	128.71 (89.64, 174.23)	126.43 (87.37, 172.18)	0.90 (-2.23, 4.13)
Turkmenistan	165.44 (119.33, 215.89)	175.7 (128.88, 230.00)	3.73 (2.29, 5.20)	181.59 (137.8, 237.81)	138.01 (94.88, 190.87)	4.48 (-5.50, 15.51)
Uzbekistan	199.10 (143.86, 262.02)	191.86 (136.61, 253.92)	1.88 (-8.57, 13.53)	165.15 (120.29, 221.19)	162.65 (118.80, 214.17)	0.76 (-3.94, 5.68)
Eastern Asia						
China	316.49 (227.21, 418.12)	282.60 (200.29, 374.13)	5.81 (-10.86, 25.59)	266.26 (170.28, 385.67)	238.38 (152.81, 342.82)	5.70 (-9.86, 23.94)
Democratic People's Republic of Korea	226.06 (163.87, 297.00)	214.06 (151.81, 285.47)	2.76 (0.90, 4.65)	208.54 (139.48, 292.91)	197.69 (130.95, 279.42)	2.71 (1.44, 3.98)
Japan	200.05 (138.17, 274.31)	182.70 (125.64, 251.02)	4.63 (-12.36, 24.92)	146.88 (94.96, 214.64)	139.34 (90.23, 203.75)	2.67 (-9.25, 16.16)
Mongolia	179.82 (130.33, 235.20)	171.57 (122.35, 227.15)	2.37 (1.63, 3.12)	138.35 (93.40, 196.70)	135.62 (93.91, 190.17)	1.02 (-5.46, 7.93)
Republic of Korea	397.91 (289.47, 514.53)	355.74 (260.07, 466.24)	5.76 (1.68, 10.01)	293.55 (193.71, 421.09)	259.26 (170.32, 371.61)	6.41 (3.63, 9.27)
Taiwan (Province of China)	182.01 (133.51, 236.40)	166.79 (122.00, 221.02)	4.47 (2.93, 6.03)	155.48 (106.42, 212.65)	145.51 (101.03, 199.77)	3.37 (0.63, 6.19)
South-eastern Asia						
Brunei Darussalam	475.04 (331.41, 645.55)	434.33 (307.58, 589.66)	4.58 (3.30, 5.87)	373.66 (265.29, 507.24)	348.36 (249.61, 475.09)	3.57 (-19.19, 32.73)
Cambodia	129.92 (92.66, 171.04)	120.72 (86.44, 160.70)	3.73 (-3.27, 11.24)	155.77 (113.20, 209.74)	149.89 (109.82, 201.55)	1.95 (-1.53, 5.54)
Indonesia	137.78 (95.52, 184.12)	60.85 (39.24, 83.94)	47.78 (-91.80, 2564.31)	141.68 (108.50, 179.92)	98.46 (79.12, 121.04)	21.04 (-64.87, 317.02)
Lao People's Democratic	162.55 (116.44, 213.01)	152.48 (110.20, 202.50)	3.25 (2.64, 3.86)	197.36 (145.05, 263.43)	191.11 (139.33, 254.27)	1.62 (1.09, 2.16)

**Republic**						
**Malaysia**	185.80 (128.94, 252.28)	187.39 (127.96, 257.68)	-0.47 (-21.19, 25.7)	175.07 (127.16, 236.30)	172.87 (124.99, 234.64)	0.65 (-14.98, 19.14)
**Myanmar**	221.85 (162.33, 290.83)	209.21 (150.21, 273.41)	2.97 (-0.61, 6.69)	296.97 (226.06, 391.24)	292.45 (221.59, 381.73)	0.77 (-0.40, 1.96)
**Philippines**	109.23 (75.73, 148.27)	98.10 (67.80, 133.19)	5.52 (-8.59, 21.82)	190.73 (157.54, 232.05)	180.14 (153.35, 212.40)	2.65 (-23.80, 38.28)
**Singapore**	315.27 (223.75, 423.15)	298.41 (209.38, 401.21)	2.79 (2.21, 3.36)	221.35 (142.95, 327.42)	208.70 (133.57, 303.22)	2.99 (-0.16, 6.24)
**Taiwan (Province of China)**	182.01 (133.51, 236.40)	166.79 (122.00, 221.02)	4.47 (2.93, 6.03)	155.48 (106.42, 212.65)	145.51 (101.03, 199.77)	3.37 (0.63, 6.19)
**Thailand**	148.99 (105.25, 197.87)	148.21 (106.34, 194.75)	0.30 (-15.24, 18.68)	200.47 (151.91, 257.71)	195.26 (152.37, 248.75)	1.34 (-4.44, 7.48)
**Timor-Leste**	151.54 (108.08, 200.92)	142.16 (100.26, 190.00)	3.25 (2.39, 4.11)	144.67 (103.43, 194.54)	137.38 (98.68, 185.34)	2.62 (-0.34, 5.66)
**Viet Nam**	104.13 (75.43, 136.19)	93.27 (67.49, 120.34)	5.63 (-16.05, 32.91)	121.79 (89.15, 164.68)	114.15 (84.21, 155.68)	3.30 (-5.23, 12.59)
Southern Asia						
**Afghanistan**	524.56 (370.57, 693.62)	492.90 (349.74, 659.25)	3.16 (1.64, 4.71)	425.85 (291.38, 590.75)	403.15 (274.95, 559.00)	2.78 (0.80, 4.80)
**Bangladesh**	325.86 (236.58, 422.60)	309.02 (228.62, 401.95)	2.69 (2.57, 2.81)	278.25 (190.78, 392.60)	265.01 (181.56, 374.43)	2.47 (-1.96, 7.10)
**Bhutan**	216.72 (156.82, 285.64)	207.96 (149.92, 272.60)	2.08 (-4.24, 8.82)	176.31 (120.36, 248.12)	169.04 (115.10, 236.14)	2.13 (-3.44, 8.01)
**India**	263.43 (186.09, 350.83)	247.08 (175.80, 328.66)	3.25 (-2.95, 9.85)	205.47 (143.14, 286.02)	194.33 (135.44, 270.85)	2.83 (-1.26, 7.08)
**Iran (Islamic Republic of)**	186.55 (125.72, 258.71)	163.78 (111.27, 226.66)	6.71 (-20.50, 43.22)	142.57 (99.49, 197.54)	130.55 (91.36, 180.05)	4.48 (-22.93, 41.65)
**Maldives**	132.78 (93.09, 181.06)	125.49 (87.48, 170.35)	2.86 (-0.69, 6.55)	127.41 (89.44, 174.32)	123.90 (87.54, 168.95)	1.40 (-10.76, 15.22)
**Nepal**	324.89 (233.49, 428.80)	295.31 (210.44, 391.49)	4.90 (-2.61, 12.98)	289.46 (198.20, 403.24)	268.34 (184.25, 374.39)	3.86 (-5.28, 13.89)
**Pakistan**	300.82 (212.44, 402.55)	298.19 (209.75, 399.96)	0.45 (-13.54, 16.69)	283.20 (203.15, 375.75)	280.56 (203.04, 375.46)	0.47 (-8.44, 10.26)
**Sri Lanka**	270.48 (187.22, 359.45)	259.63 (180.37, 344.31)	2.07 (1.34, 2.80)	277.91 (202.32, 377.58)	273.24 (200.96, 366.36)	0.81 (-21.07, 28.76)
Western Asia						
**Armenia**	161.21 (111.13, 219.79)	156.54 (112.45, 203.39)	1.48 (0.67, 2.29)	119.36 (83.55, 166.25)	120.57 (86.44, 164.74)	-0.54 (-11.62, 11.93)
**Azerbaijan**	165.56 (116.79, 219.40)	160.32 (114.15, 211.95)	1.62 (-1.02, 4.34)	124.90 (85.64, 174.03)	123.55 (84.09, 173.54)	0.56 (-8.17, 10.11)
**Bahrain**	336.28 (230.76,	317.78 (219.41,	2.88 (-8.11, 15.19)	335.96 (247.34,	327.01 (242.13,	1.36 (-1.58, 4.39)

	461.98)	433.33)		447.36)	431.54)	
**Cyprus**	173.05 (115.06, 239.07)	165.15 (108.14, 231.88)	2.38 (-3.23, 8.31)	114.51 (73.21, 168.20)	113.17 (71.97, 165.56)	0.59 (-11.24, 13.99)
**Georgia**	220.88 (156.61, 296.19)	213.00 (152.47, 283.28)	1.83 (-1.58, 5.36)	155.50 (106.27, 219.51)	155.21 (107.32, 217.40)	0.07 (-14.70, 17.40)
**Iraq**	507.49 (350.04, 691.96)	479.64 (334.23, 648.01)	2.87 (-4.38, 10.68)	403.20 (274.22, 560.73)	391.71 (270.26, 541.09)	1.45 (-5.20, 8.56)
**Israel**	154.29 (103.00, 213.40)	147.80 (101.19, 201.55)	2.16 (-1.93, 6.42)	104.97 (70.54, 149.83)	104.11 (70.93, 147.64)	0.40 (-4.79, 5.89)
**Jordan**	357.97 (246.91, 484.95)	352.84 (245.05, 469.67)	0.77 (-19.85, 26.70)	270.39 (187.26, 367.84)	264.63 (186.67, 363.57)	1.08 (-9.45, 12.84)
**Kuwait**	459.29 (320.35, 618.01)	437.24 (310.51, 576.95)	2.51 (-4.48, 10.02)	320.11 (207.36, 456.53)	303.28 (202.44, 429.33)	2.76 (-9.14, 16.22)
**Lebanon**	349.04 (241.83, 471.33)	343.16 (234.53, 467.71)	0.83 (-13.58, 17.65)	283.31 (188.15, 400.17)	272.58 (182.27, 385.93)	1.95 (-4.89, 9.28)
**Oman**	229.15 (156.27, 318.06)	177.10 (121.02, 240.01)	13.75 (13.25, 14.26)	182.12 (127.07, 250.69)	161.26 (115.50, 216.59)	6.16 (-21.16, 42.95)
**Qatar**	312.34 (211.35, 431.12)	295.37 (202.18, 403.73)	2.83 (1.22, 4.47)	255.36 (175.57, 349.70)	260.04 (186.61, 352.23)	-0.98 (-24.15, 29.26)
**Saudi Arabia**	359.02 (250.33, 486.40)	343.42 (237.58, 465.08)	2.24 (-2.85, 7.60)	291.81 (202.82, 404.75)	283.45 (195.89, 396.28)	1.46 (-4.72, 8.04)
**State of Palestine**	232.16 (161.93, 311.13)	218.60 (153.63, 294.97)	3.06 (0.18, 6.01)	202.51 (146.24, 269.01)	196.36 (142.84, 262.85)	1.55 (0.66, 2.46)
**Syrian Arab Republic**	252.28 (177.08, 337.88)	239.54 (163.34, 320.88)	2.63 (1.92, 3.34)	194.17 (133.46, 273.51)	185.58 (127.03, 260.61)	2.29 (0.19, 4.43)
**Türkiye**	189.06 (136.75, 246.90)	175.74 (128.88, 230.00)	2.60 (-0.72, 6.02)	150.65 (103.03, 209.02)	138.01 (94.88, 190.87)	0.72 (-0.50, 1.96)
**United Arab Emirates**	220.47 (151.27, 300.89)	207.89 (142.15, 284.95)	2.98 (0.25, 5.78)	159.74 (109.66, 221.45)	157.15 (109.03, 216.37)	0.86 (-27.10, 39.54)
**Yemen**	247.70 (182.11, 324.63)	112.10 (81.80, 144.62)	28.67 (-74.06, 538.13)	178.45 (118.93, 253.02)	83.37 (56.81, 117.90)	24.09 (-69.70, 408.11)

*Data shown as point estimates (95% confidence intervals)

DALYs: disability-adjusted life years; AAPC: average annual percentage change

SFigure 1. Age specific incidence and DALYs in young adult females and males from 1990 to 2021. (A) age specific incidence; (B) age specific DALYs

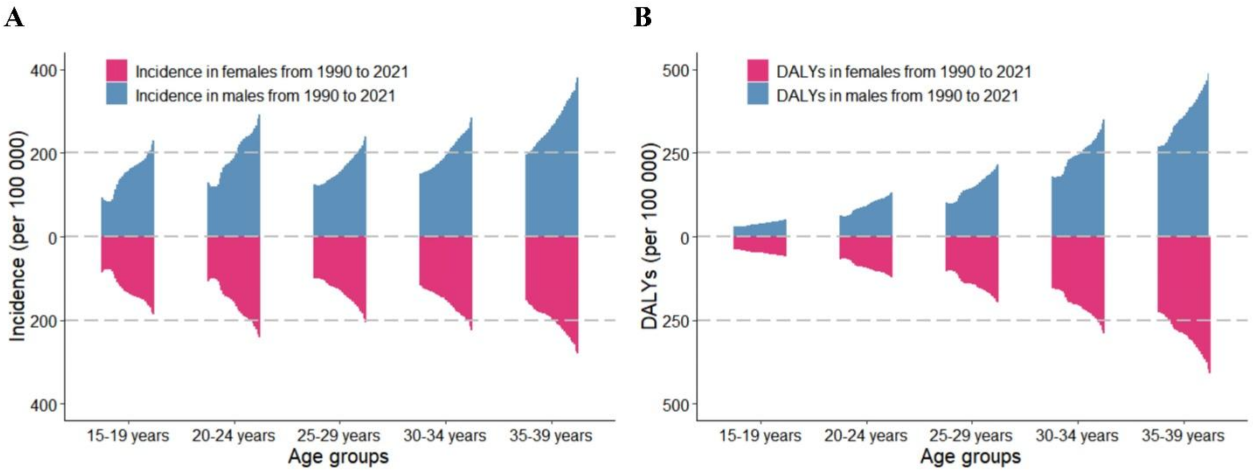

SFigure 2. Correlation between socio-demographic index (SDI) (universal health coverage [UHC], or service capacity/access) and age standardized incidence (or DALYs) of T2DM among young adults in Asia in 2021 across five age groups. (A) correlation between SDI and age standardized incidence; (B) correlation between SDI and age standardized DALYs; (C) correlation between UHC and age standardized incidence; (D) correlation between UHC and age standardized DALYs; (E) correlation between service capacity/access and age standardized incidence; (F) correlation between service capacity/access and age standardized DALYs

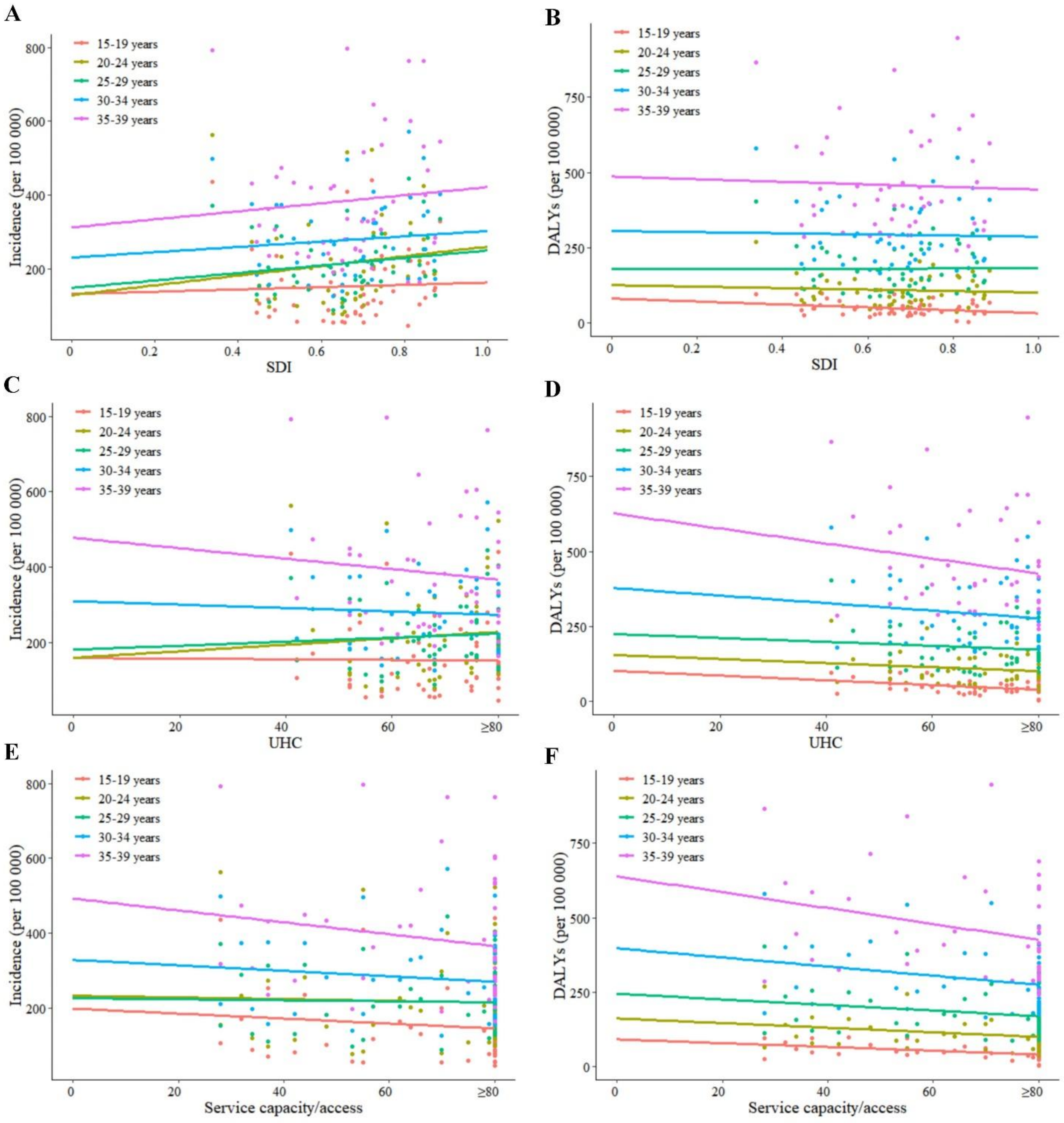

SFigure 3. Population attributable fraction (PAF) of T2DM DALYs attributable to 15 risk factors among young adults in Asia and separated by sex in 2021. (A) for both sex; (B) separated by sex

A

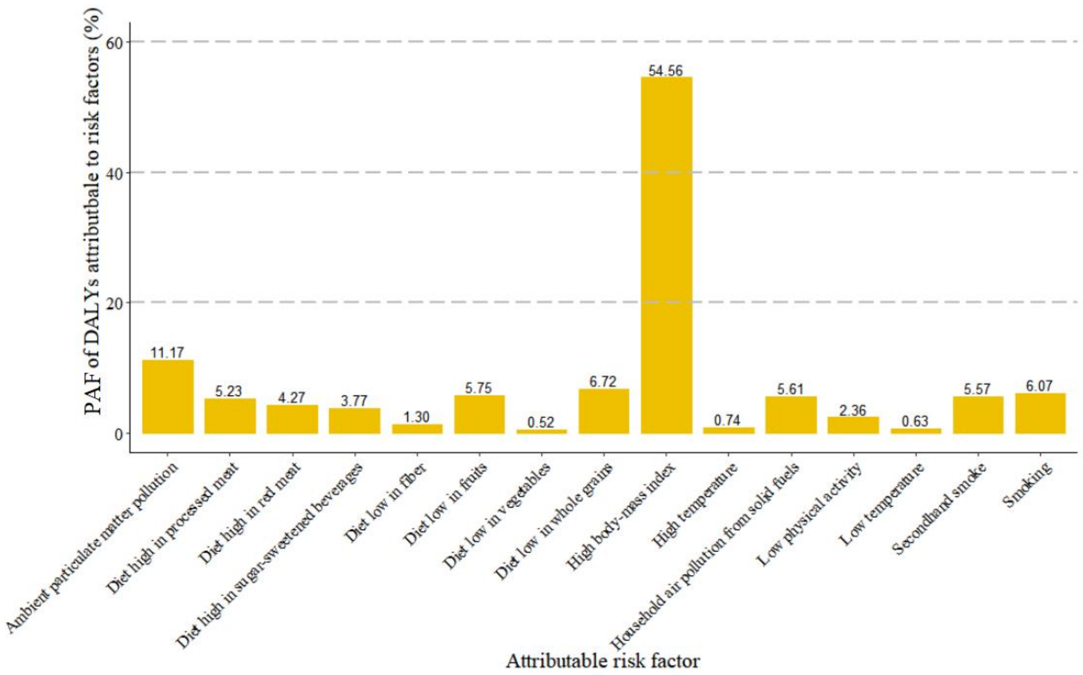

B

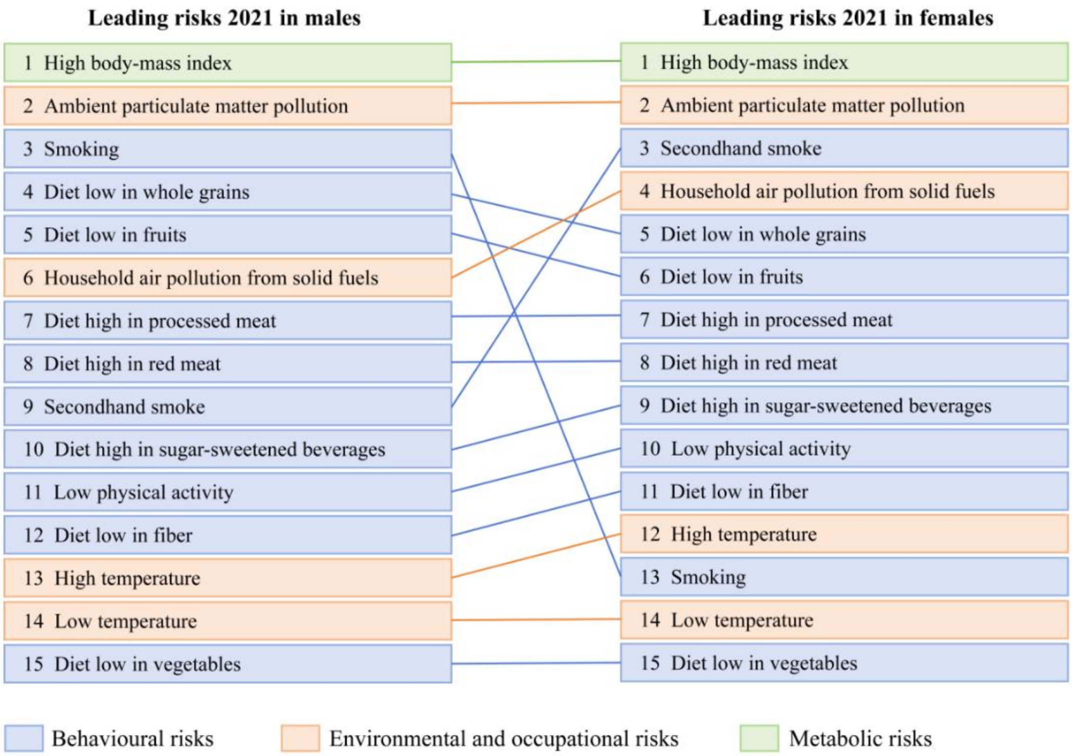

SFigure 4. Population attributable fraction (PAF) of T2DM DALYs attributable to 15 risk factors in 2021 among young adults in Asia separated by sex.

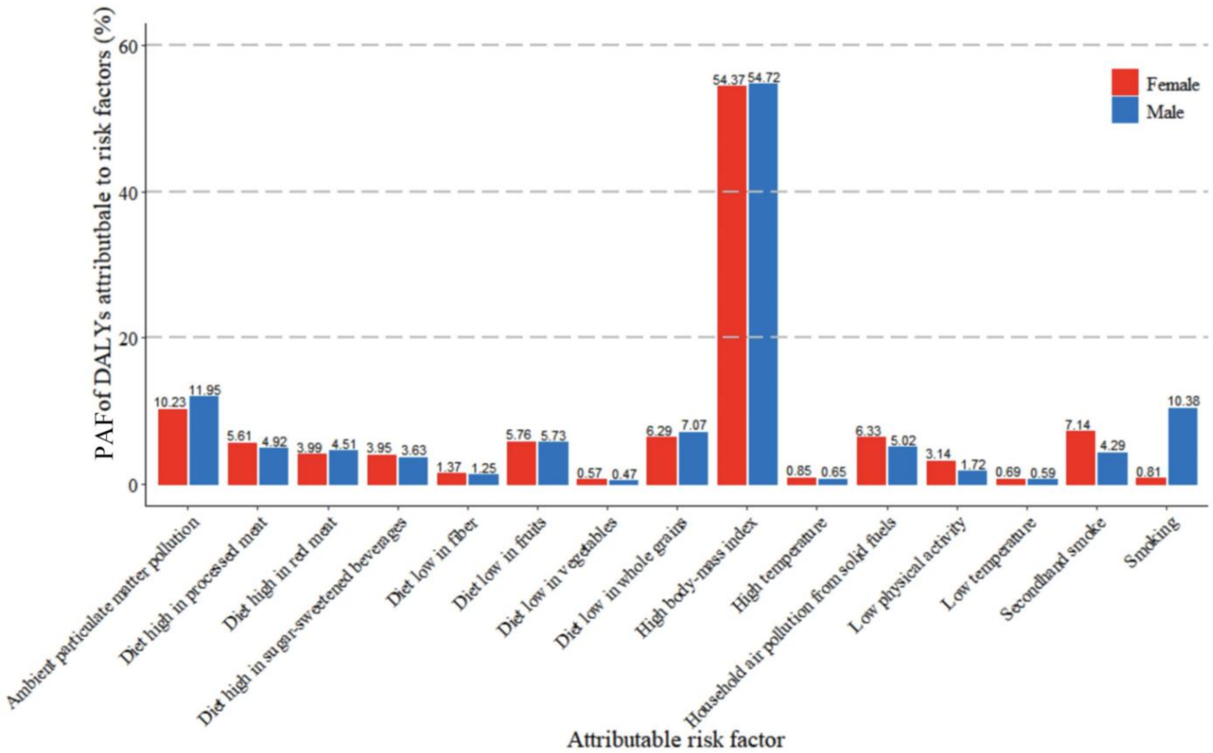

SFigure 5. Correlation between the socio-demographic index (SDI) and difference in T2DM burden between 2021 and 2019. The difference was calculated by the age standardized incidence (or DALYs) in 2021 minus those in 2019. (A) difference in age standardized incidence; (B) difference in age standardized DALYs

A

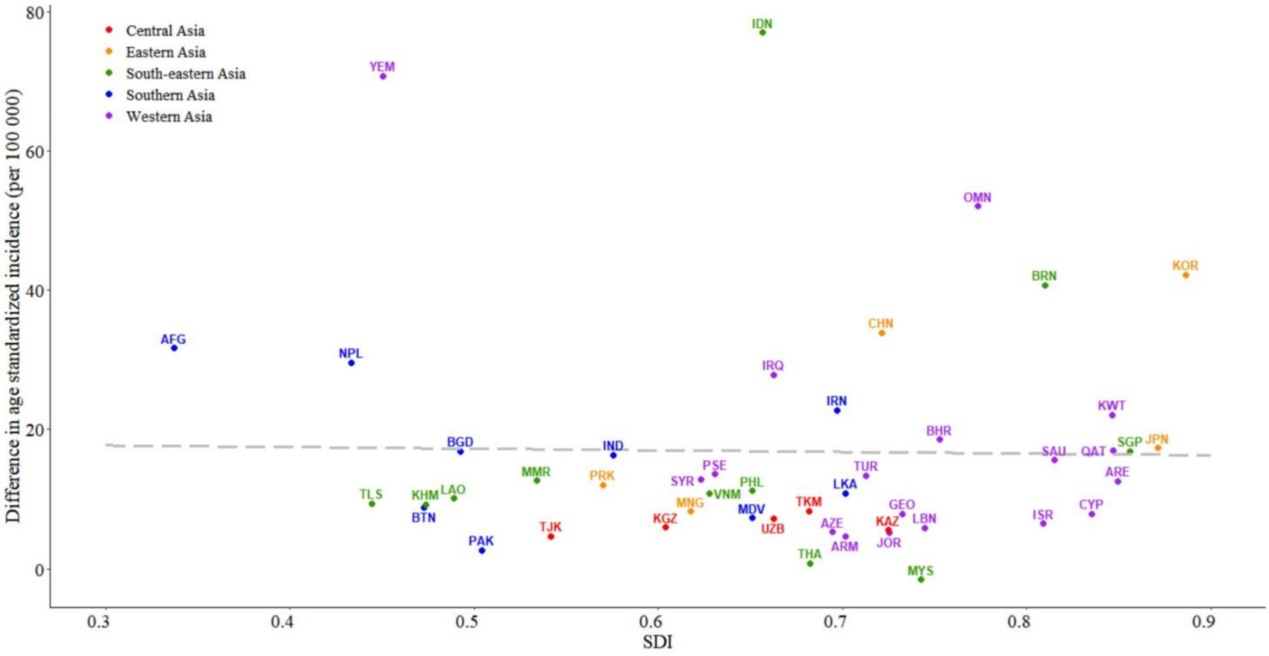

B

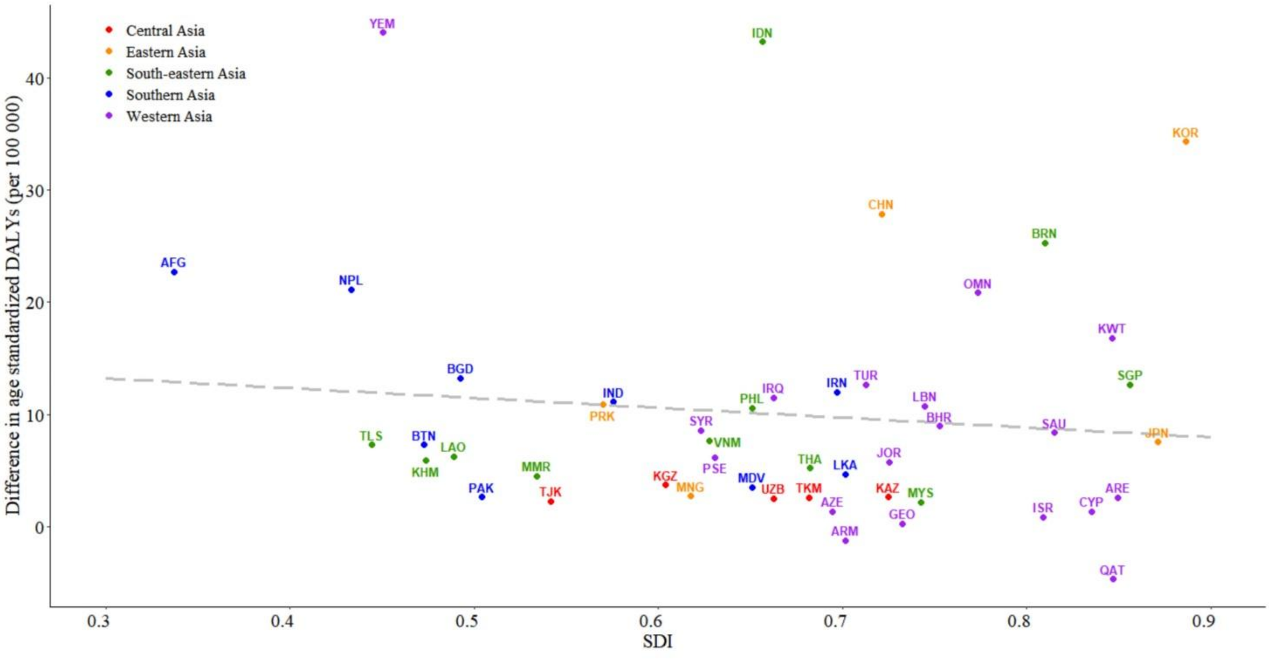

SFigure 6. Age standardized incidence and prevalence among young adults globally from 1990 to 2021 separated by different continents. (A) incidence; (B) prevalence

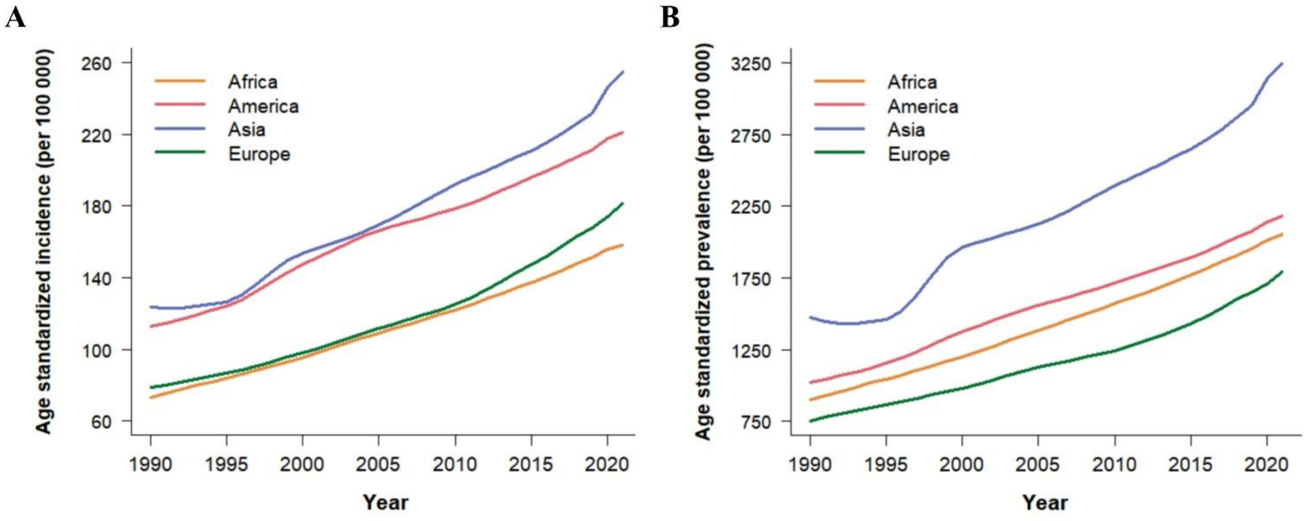

Supplement: Supporting Information 1 — Table S1: List of countries or regions in Asia as per the United Nations Statistical Division. Table S2: Age-standardized incidence, DALYs, and mortality of T2DM and AAPC among young adults in Asia∗. Table S3: Age-standardized incidence and DALYs of T2DM and AAPC among young adults by country/region in Asia∗. Table S4: AAPC for age-standardized incidence and DALYs from 1990 to 2021 among Asian young adults separated by sex and age group. Table S5: Age-standardized incidence and DALYs of T2DM and AAPC among young adults by country/region in Asia from 2019 to 2021. Figure S1: Age-specific incidence and DALYs in young adult females and males from 1990 to 2021. (A) Age-specific incidence and (B) age-specific DALYs. Figure S2: Correlation between the sociodemographic index (SDI) (universal health coverage [UHC] or service capacity/access) and age-standardized incidence (or DALYs) of T2DM among young adults in Asia in 2021 across five age groups. (A) Correlation between SDI and age-standardized incidence, (B) correlation between SDI and age-standardized DALYs, (C) correlation between UHC and age-standardized incidence, (D) correlation between UHC and age-standardized DALYs, (E) correlation between service capacity/access and age-standardized incidence, and (F) correlation between service capacity/access and age-standardized DALYs. Figure S3: Population attributable fraction (PAF) of T2DM DALYs attributable to 15 risk factors among young adults in Asia and separated by sex in 2021. (A) For both sexes and (B) separated by sex. Figure S4: Population attributable fraction (PAF) of T2DM DALYs attributable to 15 risk factors in 2021 among young adults in Asia separated by sex. Figure S5: Correlation between the sociodemographic index (SDI) and the difference in T2DM burden between 2021 and 2019. The difference was calculated by the age-standardized incidence (or DALYs) in 2021 minus those in 2019. (A) Difference in age-standardized incidence and (B) difference in age-standardiz [file 5521613.f1.pdf]
